# Supplementary material for: Sustained North Atlantic warming drove anomalously intense MIS 11c interglacial
Source: Nat Commun. 2024 Jul 15;15:5933. doi: 10.1038/s41467-024-50207-1 (PMC11251152; doi:10.1038/s41467-024-50207-1)
Supplement: Supplementary file 1 — Supplementary Information [file 41467_2024_50207_MOESM1_ESM.pdf]

Supplementary Information

Sustained North Atlantic warming drove anomalously intense MIS 11c interglacial

Hsun-Ming Hu et al.

This PDF file includes:

Supplementary Figures 1 to 10

Supplementary Table 1

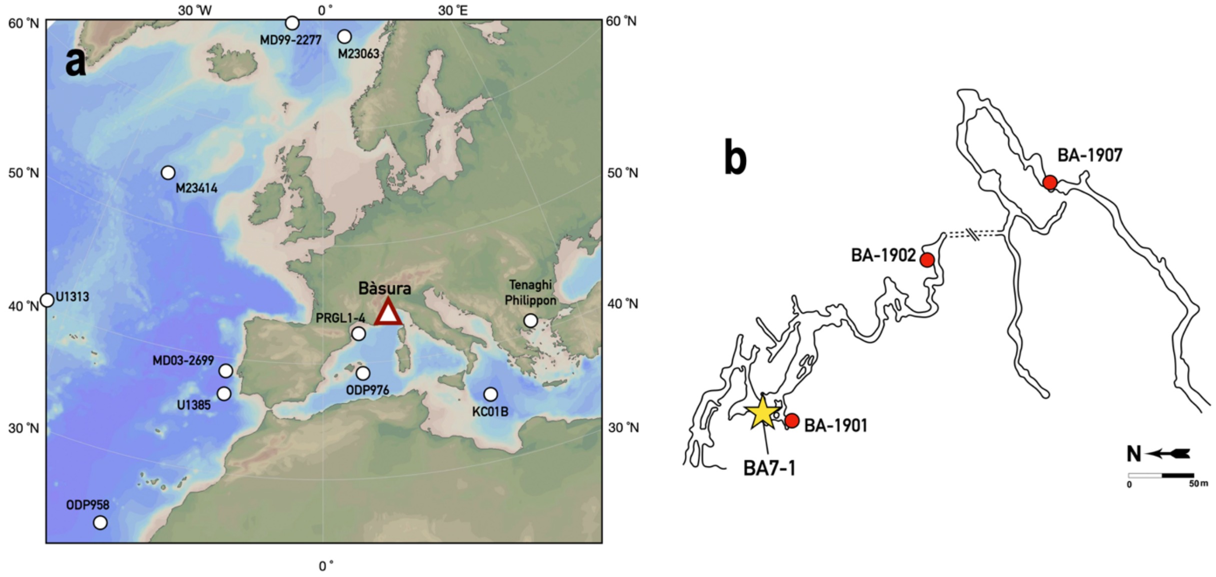

Supplementary Figure 1. Map. (a) Locations of the sites mentioned in this study. The map was generated with Ocean Data View[1]. **(b)** The interior map of Bàsura cave, with locations of flowstone BA7-1 (star), and three drip water monitoring sites BA-1901, BA-1902, and BA-1907 (red circles).

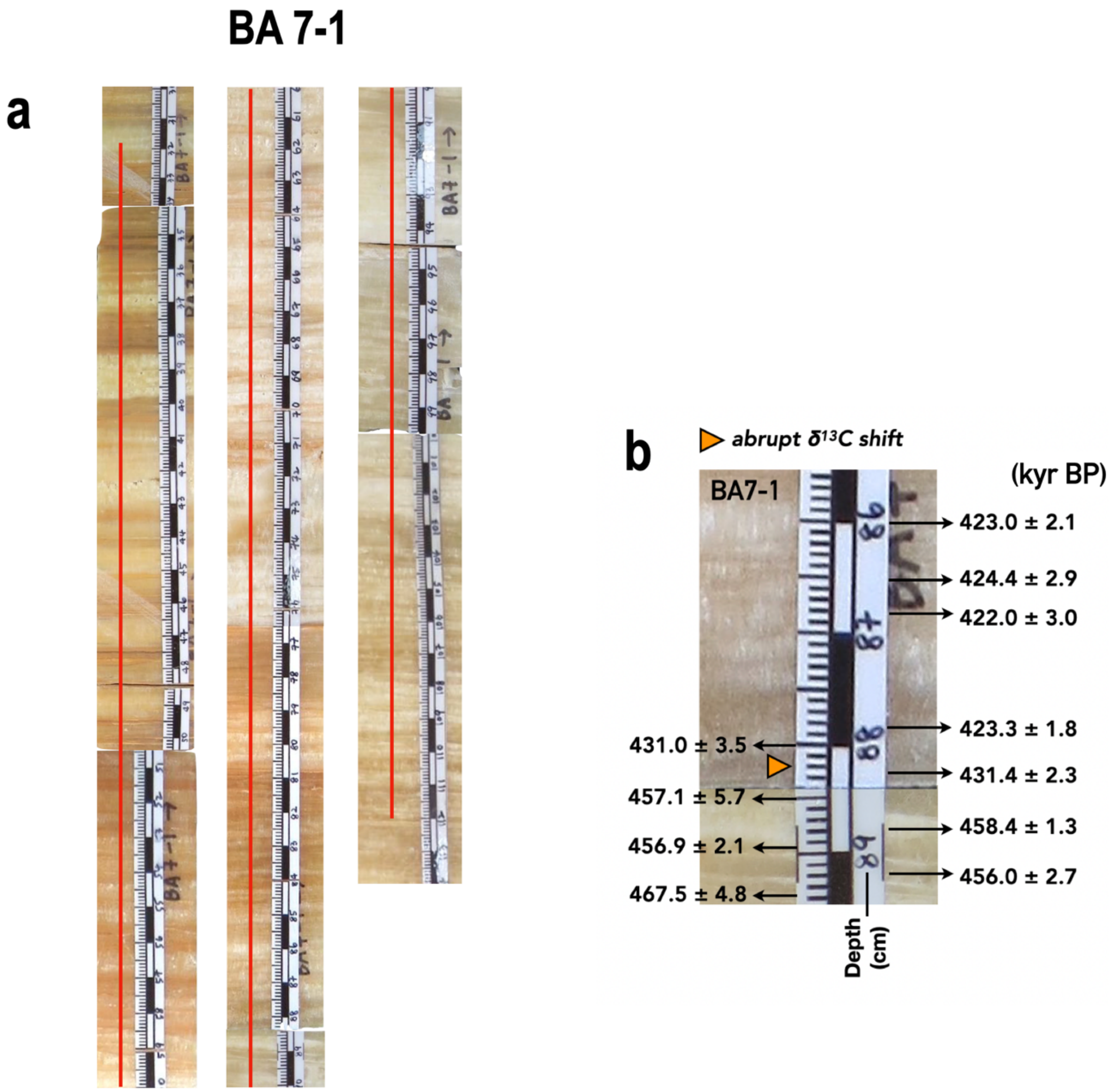

Supplementary Figure 2. Slabbed flowstone core. (a) Flowstone BA7-1 from 300 to 1,140 mm. Red lines denote the positions of subsamples for isotope and trace element analyses (317 to 1120 mm). **(b)** Enlarged BA7-1 from 855 to 895 mm with U-Th dating results and the corresponding 2σ uncertainties (in "thousand years ago"; kyr BP). The orange triangle denotes the layer with an abrupt shift in δ^{13}C. Black/white ribbon is a ruler with a cm scale.

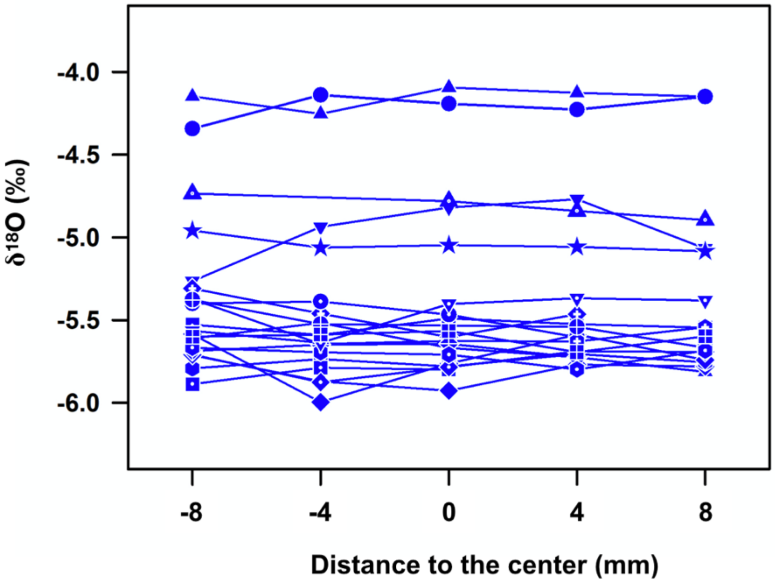

Supplementary Figure 3. Hendy test. Hendy test[2] of 14 individual layers of BA7-1 at 360, 451, 513, 540, 579, 606, 628, 675, 699, 725, 780, 820, 856 and 970 mm. The one-sigma variability of ± 0.04–0.11 ‰ in $\delta^{18}O$ of coeval subsamples from the same growth layers indicates insignificant kinetic isotope fractionation[2].

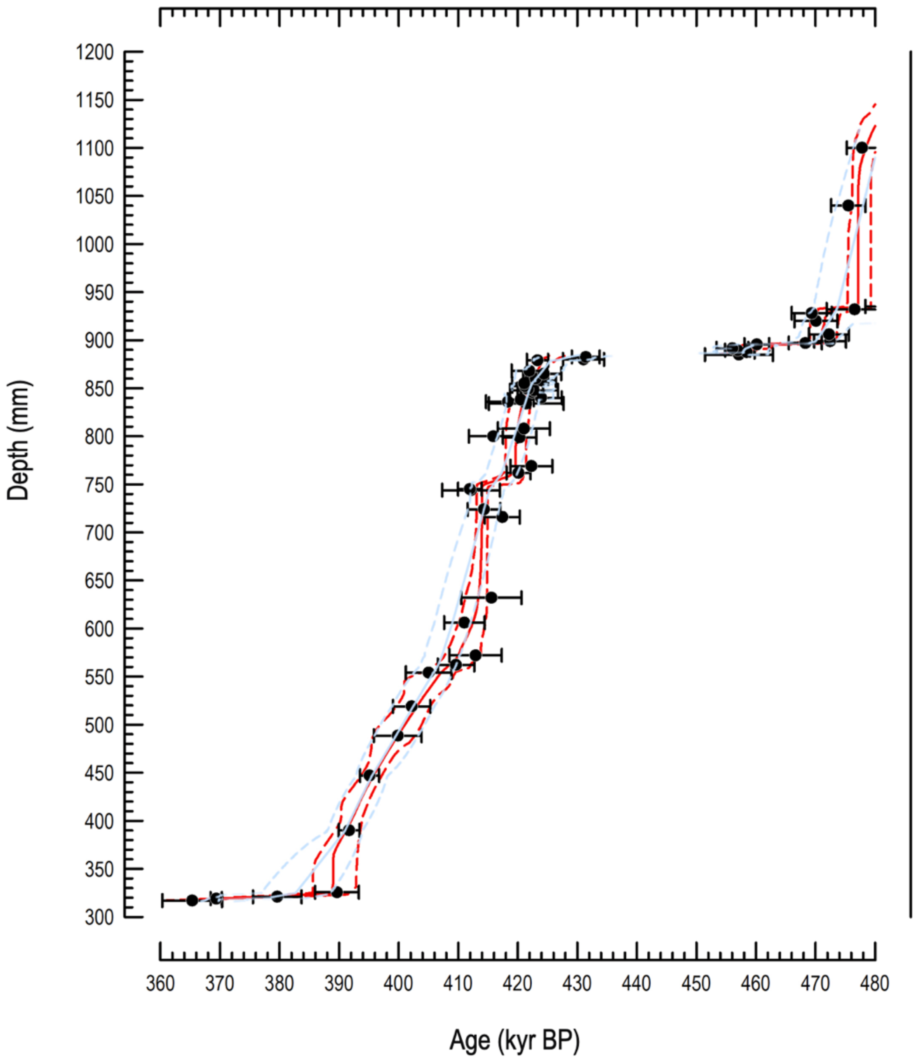

Supplementary Figure 4. Chronology of BA7-1. Red and light blue indicate age models of BA7-1 based on the StalAge[3] and OxCal[4], respectively. Dots represent ^{230}Th ages. Error bars indicate 2σ uncertainties. Solid and dashed lines denote the median model age and the 95% confidence limits of the age model, respectively.

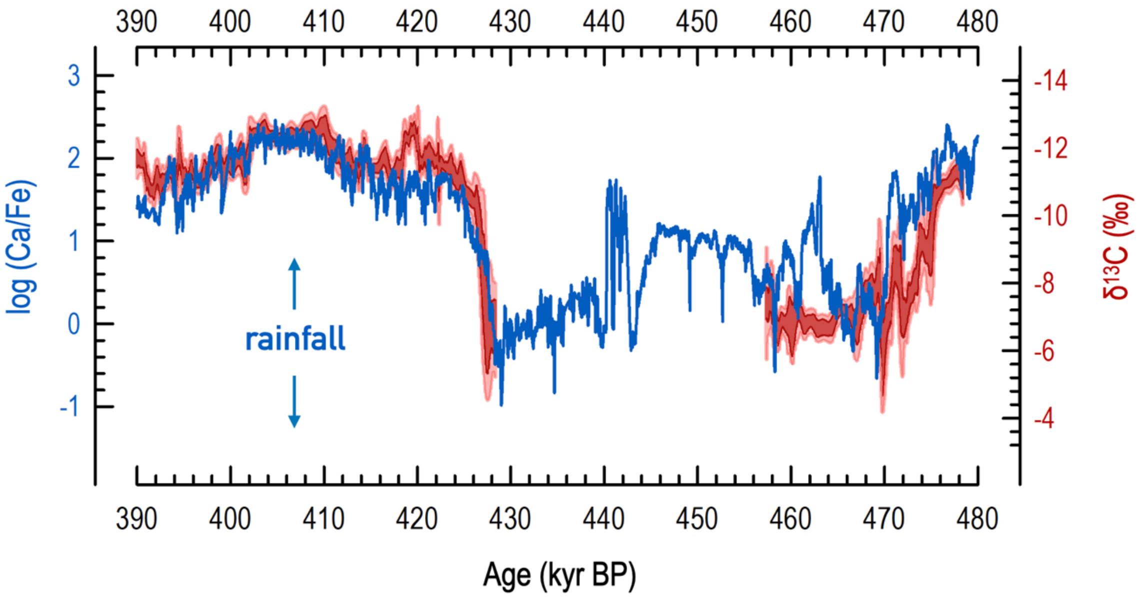

Supplementary Figure 5. Comparison between Bàsura cave δ^{13}C and rainfall reconstruction from northern Greece. Red: probabilistic analysis of Bàsura δ^{13}C using Monte Carlo simulation. The lighter and darker red shaded envelopes represent the 95% and 68% confidence intervals, respectively. Blue: log (Ca/Fe) ratio of lacustrine sediments from Tenaghi Philippon, Greece[5], whereby high values indicate high rainfall, and *vice versa*.

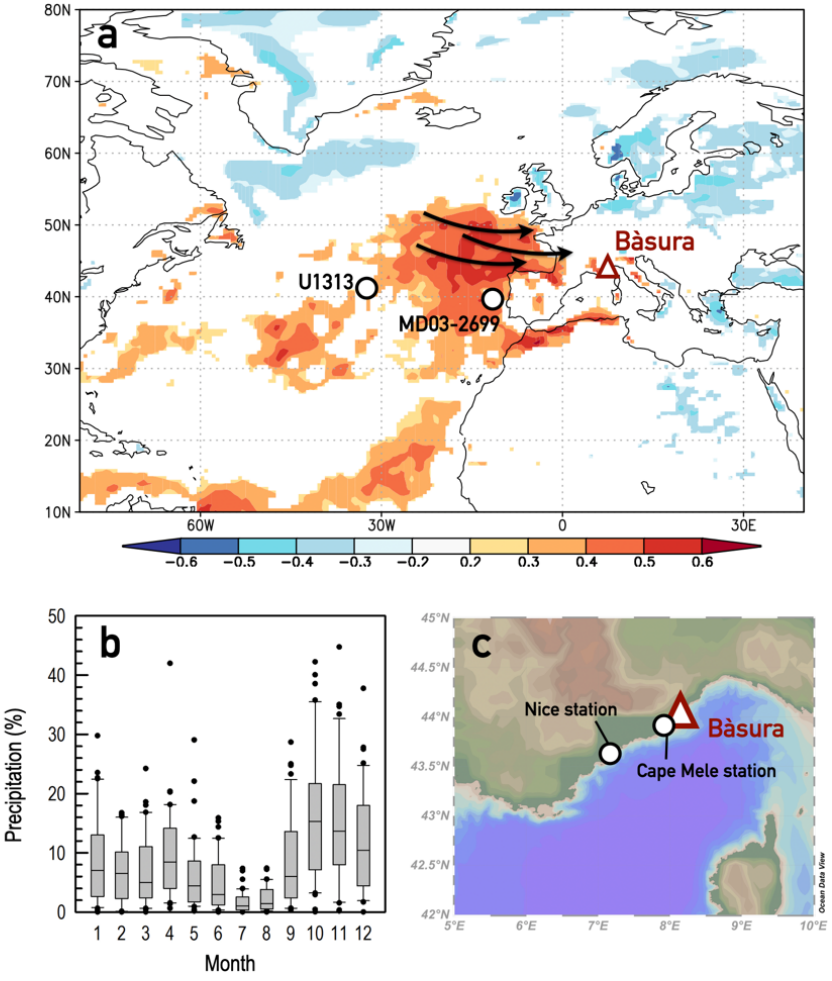

Latent Heat Fluxes vs Bàsura pp (Oct-Mar; 1980-2020 C.E.)

Supplementary Figure 6. Modern climate in the Bàsura cave region. (a) Correlation map between latent heat flux in the North Atlantic Ocean (NASA MERRA-2 global reanalysis) and precipitation amount at Bàsura cave from 1980 to 2020 C.E. (October-March). Red and blue coloured shading indicates correlation coefficients above the 90% confidence level. Locations of Bàsura cave and marine sediment archives (core MD03-2699, IODP Site U1313) are indicated, and the arrows indicate the dominant westerly wind directions based on modern reanalysis data (NCAR/NCEP 20th century reanalysis v3) during 1950-2008 C.E. in this region. The map was generated by KNMI[6]. **(b)** Monthly precipitation amount distribution at the Nice (43.65˚N, 7.21˚E, 2.0 m above sea level) and Cape Mele (43.95˚N, 8.17˚E, 221.0 m above sea level) meteorological stations between 1980 and 2020 C.E. The lower limit of the error bar indicates the minimum value in the dataset. The lower edge of the box denotes the 25th percentile, while the line inside the box represents the median. The upper edge of the box corresponds to the 75th percentile, and the upper limit of the error bar represents the maximum value. Outliers are shown as individual data points beyond the whiskers. **(c)** Locations of Bàsura cave, Nice and Cape Mele meteorological station. The map was generated by Ocean Data View[1].

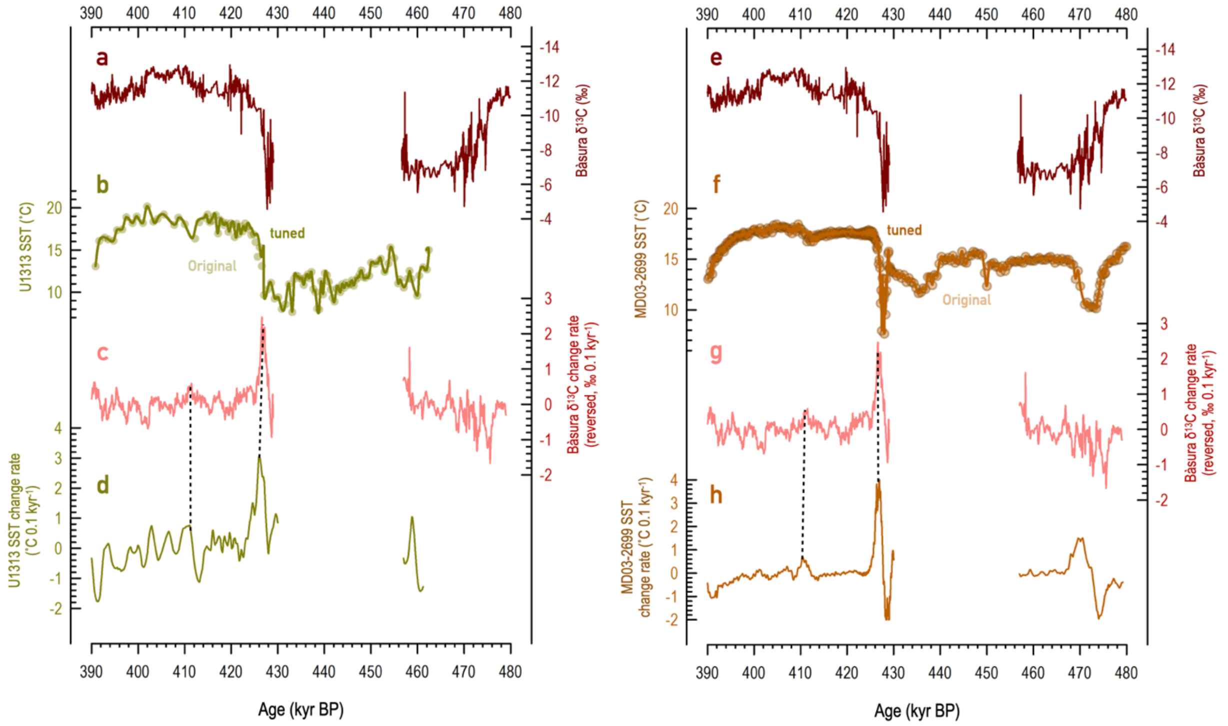

Supplementary Figure 7. Synchronisation of the U1313 and MD03-2699 with Bàsura cave records. (a) and **(e)** Bàsura δ^{13}C. **(b)** Sea-surface temperature (SST) at Site U1313 (ref. [7]). Circles and solid line indicate the original datapoints and the tuned results (this study), respectively. **(c)** and **(g)** Bàsura δ^{13}C change rate, which was calculated from 5000 Monte-Carlo simulated 1-kyr-filterd Bàsura δ^{13}C results. **(d)** and **(h)** Change rates of U1313 SST[7] and MD03-2699 SST[8], respectively. **(f)** SST at MD03-2699 (ref. [8]). Circles and solid line indicate the original datapoints and the tuned results (this study), respectively. The dashed lines in **(c)**, **(d)**, **(g)**, and **(h)** denote the tie-points from Bàsura to marine records.

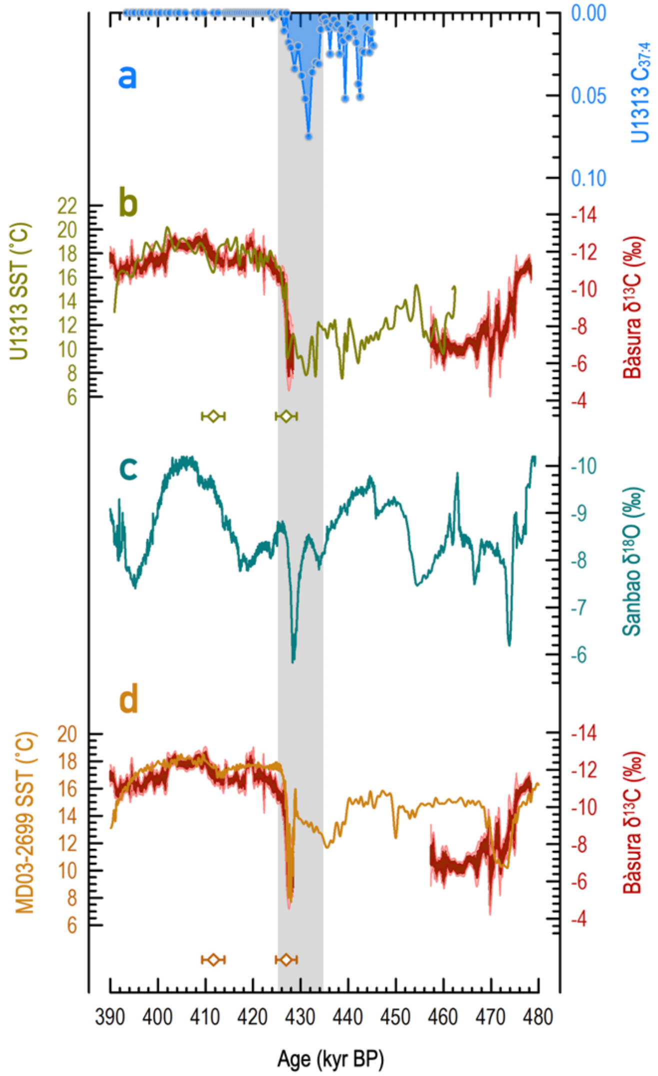

Supplementary Figure 8. Comparison of Bàsura δ^{13}C, Sanbao cave δ^{18}O, and North Atlantic time series on their radiometric chronologies. (a) Site U1313 $C_{37:4}$ (ref. [7]) on the radiometrically constrained chronology presented in this study. Higher $C_{37:4}$ indicates times of large meltwater input in North Atlantic[7]. **(b)** Red: probabilistic analysis of Bàsura δ^{13}C using Monte Carlo simulations of the data. The lighter and darker shaded envelopes represent the 95% and 68% confidence intervals, respectively. Olive: alkenone-based sea-surface temperature (SST)[7] from Site U1313 on the radiometrically constrained chronology presented in this study. Olive diamonds and error bars denote the tie points with 2σ uncertainties used to tune Site U1313 to Bàsura cave δ^{13}C. **(c)** Stalagmite δ^{18}O from Sanbao cave (eastern China), whereby low δ^{18}O indicates strong Asian monsoon intensity, and *vice versa*[9]. **(d)** Red: probabilistic analysis of Bàsura δ^{13}C using Monte Carlo simulations of the data. Bronze: alkenone-based SST[8] from core MD03-2699 on the radiometrically constrained chronology presented in this study. Bronze diamonds and error bars denote the tie points with 2σ uncertainties used to tune MD03-2699 to the Bàsura cave δ^{13}C. Grey vertical bar marks the Heinrich-like stadial associated high meltwater fluxes into North Atlantic during Termination V.

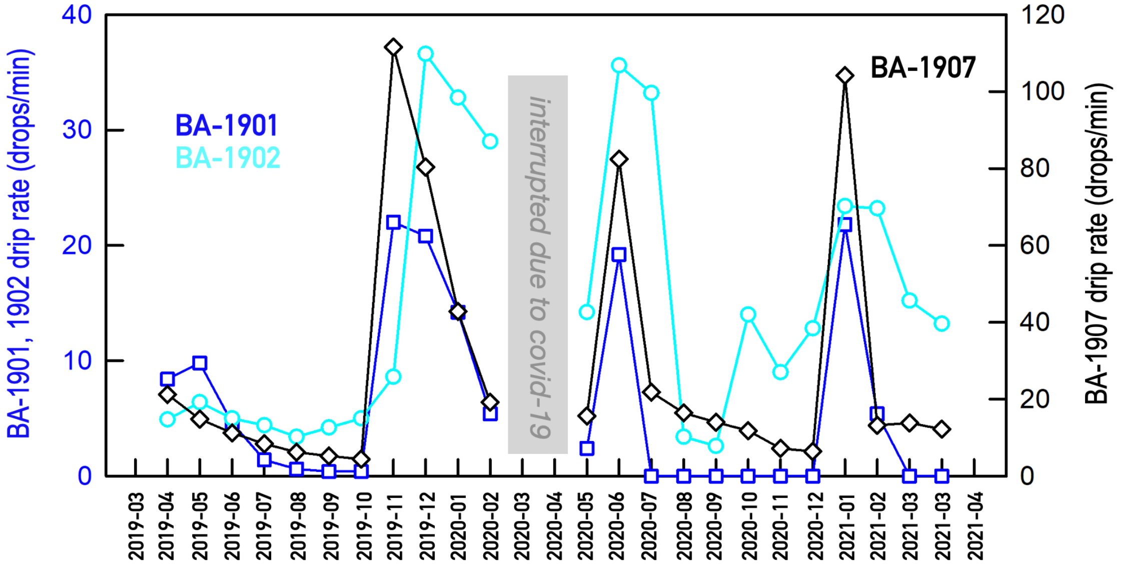

Supplementary Figure 9. Drip rates measured at Site BA-1901, BA-1902, and BA-1907. The monitoring period was from April 2019 to March 2021, with a 2-months (February and March 2020) interruption due to COVID-19 pandemic. The locations of these sites are illustrated in Supplementary Figure 1b.

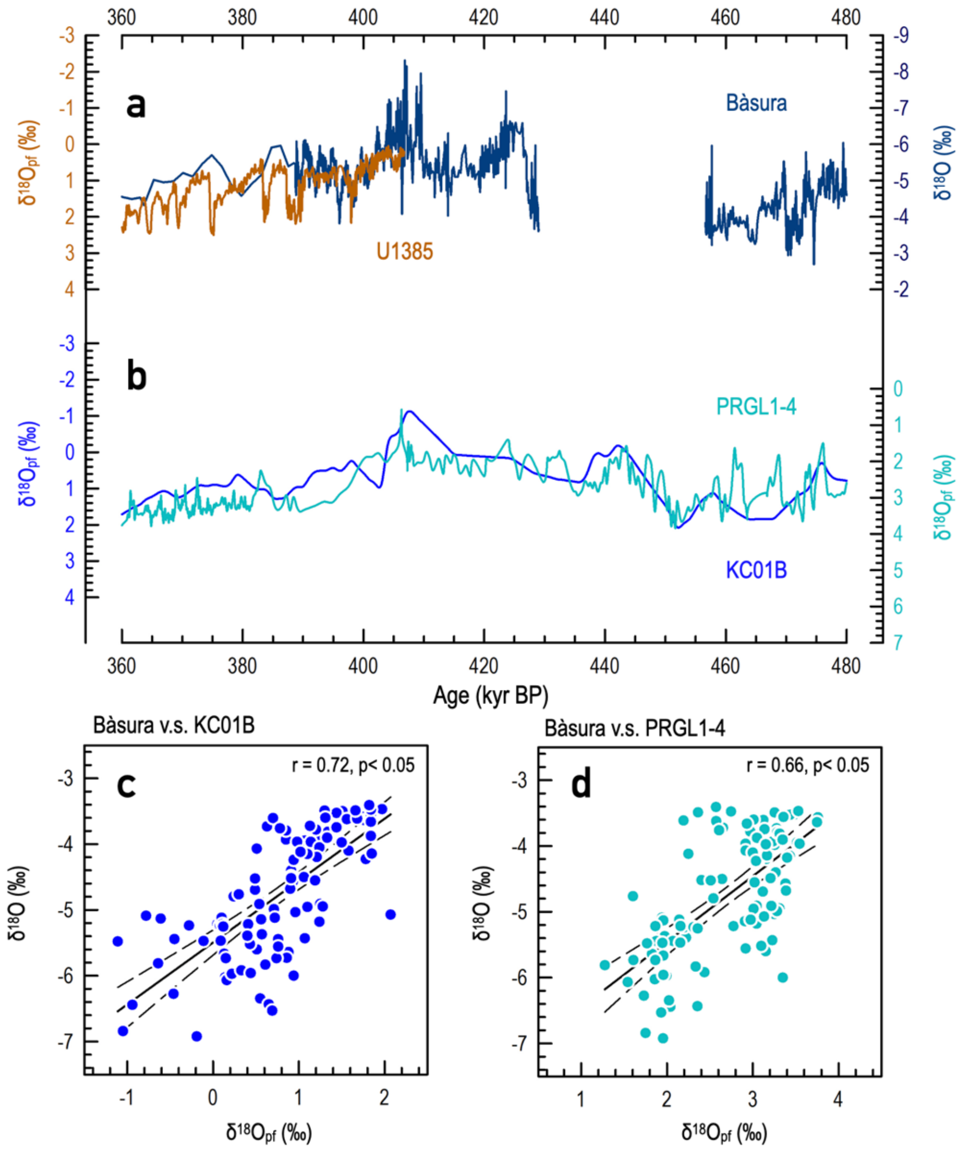

Supplementary Figure 10. Comparison between Bàsura cave δ^{18}O and planktonic foraminiferal δ^{18}O from Atlantic and Mediterranean marine sediment archives. (a) Navy blue: Bàsura cave (BA7-1) δ^{18}O time series (this study). Brown: Planktonic foraminiferal δ^{18}O ($\delta^{18}O_{pf}$)[10] of Site U1385 on the radiometrically constrained chronology presented in this study. **(b)** $\delta^{18}O_{pf}$ time series for core KC01B (ref. [11]) and PRGL1-4 (ref. [12]) on their original chronologies. **(c)** cross-plot and linear correlation analysis between 1-kyr-resampled Bàsura cave δ^{18}O and $\delta^{18}O_{pf}$ from KC01B. **(d)** cross-plot and linear correlation analysis between 1-kyr-resampled Bàsura cave δ^{18}O and $\delta^{18}O_{pf}$ from PRGL1-4. Correlation coefficients (r) and p values are also shown.

Supplementary Table 1. Data of change point determination.

Record	Interval (kyr BP)	Algorithm	Age (kyr BP)	Change point 2σ (kyr)	Age model 2σ (kyr)	RMS* 2σ (kyr)
U1313 sea-surface temperature (SST)	400–440	RAMPFIT[13]	424.5	1.4	4.4	4.6
	405–455	RAMPFIT[13]	425.0	1.9	4.4	4.8
	410–445	RAMPFIT[13]	425.5	1.4	4.4	4.6
	†415–435	RAMPFIT[13]	425.0	1.2	4.4	4.6
	420–450	RAMPFIT[13]	425.5	1.4	4.4	4.6
	420–440	RAMPFIT[13]	425.5	1.7	4.4	4.7
MD03-2699 SST	400–440	RAMPFIT[13]	426.4	0.8	4.4	4.5
	405–455	RAMPFIT[13]	426.1	1.3	4.4	4.6
	410–445	RAMPFIT[13]	426.2	1.3	4.4	4.6
	†415–435	RAMPFIT[13]	426.4	1.2	4.4	4.6
	420–450	RAMPFIT[13]	426.2	2.0	4.4	4.8
	420–440	RAMPFIT[13]	426.4	1.5	4.4	4.7
Bàsura δ^{13}C	400–429	BREAKFIT[14]	423.0	0.1	1.2	1.2
	405–429	BREAKFIT[14]	423.0	0.1	1.3	1.3
	410–429	BREAKFIT[14]	422.8	0.1	1.2	1.2
	†415–429	BREAKFIT[14]	423.1	0.1	1.3	1.3
	420–429	BREAKFIT[14]	423.4	0.1	1.4	1.4

*Root-mean-square errors.
†Reported data in main text.

References

1. Schlitzer, R. (2024). Ocean Data View, http://odv.awi.de.
2. Hendy, C. (1971). The isotopic geochemistry of speleothems—I. The calculation of the effects of different modes of formation on the isotopic composition of speleothems and their applicability as palaeoclimatic indicators. *Geochimica et Cosmochimica Acta*, *35*(8), 801–824. https://doi.org/10.1016/0016-7037(71)90127-X.
3. Scholz, D., & Hoffmann, D. L. (2011). StalAge – An algorithm designed for construction of speleothem age models. *Quaternary Geochronology*, *6*(3), 369–382. https://doi.org/https://doi.org/10.1016/j.quageo.2011.02.002
4. Ramsey, C. B. (2008). Deposition models for chronological records. *Quaternary Science Reviews*, *27*(1), 42–60. https://doi.org/https://doi.org/10.1016/j.quascirev.2007.01.019
5. Koutsodendris, A. et al. (2023). Atmospheric CO_2 forcing on Mediterranean biomes during the past 500 kyrs. *Nature Communications*, *14*(1), 1664. https://doi.org/10.1038/s41467-023-37388-x
6. U.S. Federal Government, 2023: U.S. Climate Resilience Toolkit Climate Explorer. https://crt-climate-explorer.nemac.org/ (Accessed on June 13, 2024)
7. Stein, R., Hefter, J., Grützner, J., Voelker, A., & David A Naafs, B. (2009). Variability of surface water characteristics and Heinrich-like events in the Pleistocene midlatitude North Atlantic Ocean: biomarker and XRD records from IODP site U1313 (MIS 16-9). *Paleoceanography*, *24*(2), 1–13. https://doi.org/10.1029/2008PA001639
8. Rodrigues, T., Voelker, A. H. L., Grimalt, J. O., Abrantes, F., & Naughton, F. (2011). Iberian margin sea surface temperature during MIS 15 to 9 (580-300 ka): glacial suborbital variability versus interglacial stability. *Paleoceanography*, *26*(1), 1–16. https://doi.org/10.1029/2010PA001927
9. Cheng, H. et al. (2016). The Asian monsoon over the past 640,000 years and ice age terminations. *Nature*, *534*(7609), 640–646. https://doi.org/10.1038/nature18591
10. Nehrbass-Ahles, C. et al. (2020). Abrupt CO_2 release to the atmosphere under glacial and early interglacial climate conditions. *Science*, *369*(6506), 1000–1005. https://doi.org/10.1126/SCIENCE.AAY8178
11. Wang, P., Tian, J., & Lourens, L. J. (2010). Obscuring of long eccentricity cyclicity in Pleistocene oceanic carbon isotope records. *Earth and Planetary Science Letters*, *290*(3–4), 319–330. https://doi.org/10.1016/j.epsl.2009.12.028
12. Frigola, J. et al. (2012). A 500 kyr record of global sea-level oscillations in the Gulf of Lion, Mediterranean Sea: New insights into MIS 3 sea-level variability. *Climate of the Past*, *8*(3), 1067–1077. https://doi.org/10.5194/cp-8-1067-2012
13. Mudelsee, M. (2000). Ramp function regression: a tool for quantifying climate transitions. *Computers & Geosciences*, *26*(3), 293–307. https://doi.org/https://doi.org/10.1016/S0098-3004(99)00141-7
14. Mudelsee, M. (2009). Break function regression. *The European Physical Journal Special Topics*, *174*(1), 49–63. https://doi.org/10.1140/epjst/e2009-01089-3
