# Supplementary material for: Sustained North Atlantic warming drove anomalously intense MIS 11c interglacial
Source: Nat Commun. 2024 Jul 15;15:5933. doi: 10.1038/s41467-024-50207-1 (PMC11251152; doi:10.1038/s41467-024-50207-1)
Supplement: Supplementary file 2 — Description of Additional Supplementary Files [file 41467_2024_50207_MOESM2_ESM.pdf]

Description of Additional Supplementary Files

Supplementary Data 1:
$\delta^{18}O$, $\delta^{13}C$ and growth rate of BA7-1

Supplementary Data 2:
Sr/Ca of BA7-1

Supplementary Data 3:
U-Th dating results of BA7-1

Supplementary Data 4:
Tuned records of U1313 and MD03-2699
